# Knockdown of NOLC1 Inhibits PI3K-AKT Pathway to Improve the Poor Prognosis of Esophageal Carcinoma

**DOI:** 10.1155/2021/9944132

**Published:** 2021-05-08

**Authors:** Fanguo Kong, Yansheng Shang, Xingyuan Diao, Jiaguo Huang, Hui Liu

**Affiliations:** ^1^Department of Gastroenterology, Jinan City People's Hospital, Jinan People's Hospital Affiliated to Shandong First Medical University, Jinan 271199, China; ^2^Department of Gastroenterology, Shandong Provincial Hospital Affiliated to Shandong First Medical University, Jinan 271199, China

## Abstract

**Objective:**

Esophageal carcinoma (ESCA) is a common malignant gastrointestinal tumor. The abnormal expression of NOLC1 is involved in the tumorigenesis of various human tumors, whereas the function and mechanism of NOLC1 in ESCA remain unclear. In this study, we explored the relationship between NOLC1 and poor prognosis of ESCA, and its role and mechanism in the occurrence of ESCA.

**Methods:**

The NOLC1 expression in ESCA tissues and cell lines was determined by qRT-PCR, immunohistochemistry, or western blot. The Kaplan–Meier method was conducted to estimate the overall survival. Cox regression analysis was carried out to examine the association between patient characteristics and prognosis. A recombined lentiviral vector containing NOLC1 was applied for transfecting ESCA cells (Eca109 and TE-13) and established a stable cell line with low NOLC1 expression or high NOLC1 expression, in the absence or presence of PI3K inhibitor (LY294002) treatment. Cell proliferation, apoptosis rate, invasion ability, migration ability, and PI3K/AKT pathway were detected by CCK8 assay, flow cytometry, Transwell assay, wound-healing assay, and western blot.

**Results:**

NOLC1 overexpression was observed in ESCA tissues and ESCA cell lines (EC9706, Eca109, TE-13, Kyse170, T.TN) compared with adjacent normal tissues and normal esophageal cell line HEEC. NOLC1 overexpression was markedly associated with bigger tumor size, lymph node metastasis, and advanced TNM stage. Patients with NOLC1 overexpression have shorter overall survival than that of those with low NOLC1 expression. NOLC1 overexpression was considered to be an independent poor prognostic factor affecting overall survival. NOLC1 knockdown inhibited proliferation, migration, invasion, and cyclin B1 expression and promoted the apoptosis and cleaved-caspase-3 expression of Eca109 and TE-13 cells. NOLC1 overexpression accelerated proliferation, migration, invasion, and cyclin B1 expression and inhibited the apoptosis and cleaved-caspase-3 expression of ESCA cells via activating PI3K/AKT pathway. Rescue experiments showed that PI3K inhibitor (LY294002) could reverse the phenomenon caused by NOLC1 overexpression.

**Conclusion:**

NOLC1 may be a marker for poor prognosis. It can participate in the occurrence and development of ESCA via the PI3K/AKT pathway.

## 1. Introduction

Esophageal carcinoma (ESCA) is a common malignant gastrointestinal tumor, which is the sixth leading cause of mortality among all kinds of tumors worldwide. Because the incidence of ESCA is relatively insidious and the early clinical symptoms are not typical, the majority of patients are already in the middle and advanced stages of treatment [1, 2]. Although resection operation is currently the main therapeutic schedule, comprehensive treatment including chemotherapy and radiotherapy is difficult to provide a survival benefit and improved quality of life. The 5-year survival rate of patients with ESCA remain poor, <20%, and the recurrence rate is more than 40% [3]. The cause of death is tumor recurrence or metastasis in patients with ESCA. However, the etiology and pathogenesis of ESCA remain ambiguous, which may be mediated by the cumulative effect of multiple genes [4, 5]. Therefore, it is urgently required to develop effective biomarkers for diagnosing, and new treatment strategies, such as targeted therapy, for preventing malignancy in patients with ESCA.

Nucleolar and coiled-body phosphoprotein 1 (NOLC1), a phosphoprotein, consists of a unique central repeat domain, and C-terminal and N-terminal domains [6–8]. Huang et al. reported that NOLC1 was positively correlated with the tumorigenesis of non-small cell lung cancer (NSCLC) and might be utilized as biomarkers for the early diagnosis of NSCLC [9]. Additionally, Hwang et al. showed that NOLC1 cooperates with TP53 synergistically to activate a cellular proto-oncogene MDM2, leading to cell growth and impediment of apoptosis in nasopharyngeal carcinoma [7, 10], whereas the function and mechanism of NOLC1 in the occurrence of ESCA remain unknown.

In the current study, we analyzed the gene expression profiling interactive analysis (GEPIA) database and discovered that, compared with normal esophageal epithelial tissues, ESCA tissues tend to have a relatively higher of NOLC1 expression. Thus, we measured NOLC1 expression in ESCA tissues and investigated the clinical and prognostic significance of NOLC1 expression in patients with ESCA, and a transfected recombinant lentiviral vector containing NOLC1 into ESCA cell lines with or without PI3K inhibitor (LY294002), to further investigate the function and mechanism of NOLC1 expression in ESCA.

## 2. Methods

### 2.1. Clinical Specimens

In this study, a total of 45 patients with ESCA who underwent surgical resection were recruited between January 2010 and December 2015 from Jinan City People's Hospital, Jinan People's Hospital Affiliated to Shandong First Medical University, Jinan City, Shandong Province, China. And 19 adjacent normal tissue specimens 3 cm away from the edge of the primary tumor were excised as control. In all cases, the diagnosis of each clinical specimen was verified by two pathologists at Jinan City People's Hospital. International Union Against Cancer (UICC) guidance was utilized for tumor staging. Gene expression profiling of interactive analysis website was carried out to analyze NOLC1 expression in ESCA tissues and normal tissues. The clinical parameters from the patients' medical records are analyzed in Table 1. All patients signed informed consent, and the ethics committee of Jinan City People's Hospital approved this study.

### 2.2. QRT-PCR

Total RNA was obtained from tissue specimens and cells using TRIzol (Thermo Fisher, Shanghai, China). The purity and concentration of RNA samples were determined using spectrophotometry (BioTek, Vermont, USA). Subsequently, 300 ng RNA samples were reverse-transcribed into cDNAs and amplificated by using a One Step SuperRT-PCR Mix Kit (Solarbo, Shanghai, China). GAPDH was utilized as an expression control. NOLC1 mRNA levels were analyzed with the 2^−ΔΔCt^ method. Fold changes were calculated for Ct values of amplified NOLC1 mRNA compared with those of GAPDH.

1. NOLC1 primer: F 5′-AGCTGGCCTGACGGTATG-3′, R 5′-TTGGTCTGGCTGAGTACCG-3′
2. GAPDH primer: F 5′-TCACCAGGGCTGCTTTTA-3′, R 5′-TTCACACCCATGACGAACA-3′

### 2.3. Immunohistochemical Staining

Routine immunohistochemical staining of esophageal cancer biopsy specimens was performed using an anti-NOLC1 monoclonal antibody. Specimens embedded in paraffin wax were cut onto slides. Subsequently, slides were dewaxed, hydrated, and stained with the 3,3′-diaminobenzidine (DAB). Additionally, slides were counterstained with hematoxylin. The number of NOLC1-positive and the number of NOLC1-negative ESCA cells were counted at a magnification of ×400 under a light microscope. Cells on the section with brown nucleoli were considered positive for NOLC1. Positive scores were divided into 4 groups: “0” (no expression), “1+” (weak staining, only one nucleolus staining), “2+” (moderate staining, multiple nucleolar staining), and “3+” (strong staining, cell nuclei and nucleoli staining).

### 2.4. Western Blot

Tissues and cells were lysed in RIPA lysis buffer to isolate protein (Beyotime, Shanghai, China). Subsequently, samples were separated using 10% SDS-PAGE, transferred to PVDF membranes, and blocked in 5% BSA for 1 h. Membranes were incubated with primary antibodies, including rabbit monoclonal antibody NOLC1 (1 : 1000, Abcam, CA, USA), cleaved-caspase-3 (1 : 1000, Abcam), cyclin B1 (1 : 1000, Abcam), AKT (1 : 1000, Abcam), PI3K (1 : 1000, Abcam), p-PI3K (1 : 1000, Cell Signaling Technology, Shanghai, China), p-AKT (1 : 1000, Abcam), and rabbit polyclonal antibody GAPDH (1 : 10000, Abcam) overnight at 4°C. Membranes were washed 3 times and labeled with the HRP conjugated secondary antibodies (1 : 2000, Abcam) at room temperature for 1 h. Protein bands were visualized using an ECL system (Tannon, Beijing, China).

### 2.5. Cell Culture and Lentivirus Infection

Normal esophageal cell line HEEC and five esophageal cancer cell lines (EC9706, Eca109, TE-13, Kyse170, T.TN) were obtained from Cell Resource Center, Shanghai Institute for Biological Sciences, Chinese Academy of Sciences. All cells were cultured in RPMI-1640 medium (Gibco, Rockville, USA) containing 10% FBS (Gibco, Rockville, USA) at 37°C and 5% CO_2_ incubator. The NOLC1 overexpression and knockdown lentiviral vector were purchased from GenePharma (Shanghai, China). The cells were transduced with lentiviral vectors at a multiplicity of infection (MOI) of 10 in a serum-free medium for 12 h. Subsequently, the cells were harvested and cultured with a complete medium for further study.

### 2.6. CCK8 (Cell Counting Kit-8) Assay

CCK8 assay was carried out to examine the cell viability. After lentivirus infection, Eca109 and TE-13 cells (2 × 10^3^ cells/well) with or without PI3K inhibitor (LY294002) were resuspended and plated in 96-well plates for 0-, 24-, 48-, and 72 h incubation. Subsequently, 10 *μ*L of CCK8 solution (Beyotime, Shanghai, China) was added, dropwise, into each well and incubated for 2 h. The absorbance at a wavelength of 450 nm was measured using a microplate reader (BioTek, Vermont, USA).

### 2.7. Flow Cytometry

After lentivirus infection, Eca109 and TE-13 cells with or without LY294002 were harvested and stained with Annexin V-FITC/PI (BD Biosciences, Bedford, USA) in the dark for 15 min. Cell apoptosis was analyzed using flow cytometry.

### 2.8. Invasion Assay

Transwell chamber was applied to measure the cell invasion ability. Matrigel matrix (Becton, Dickinson and Company, Bedford, USA) was diluted with serum-free medium at a ratio of 1 : 10. Then, Matrigel was evenly added to the upper chamber. After lentivirus infection, 5 × 10^4^ Eca109 and TE-13 cells with or without LY294002 in 200 *μ*L of serum-free medium were added to the apical chamber, and 600 *μ*L of medium containing 15% FBS was supplied to the bottom chamber for 48 h. Cells in apical chamber were wiped off, fixed with 5% paraformaldehyde, and then stained with 0.1% crystal violet solution for 15 minutes. Finally, cell invasion was evaluated by counting under the microscope field (Olympus, Tokyo, Japan).

### 2.9. Wound-Healing Assay

Wound-healing assay was carried out to detect cell migration. After lentivirus infection, Eca109 and TE-13 cells (5 × 10^5^ cells) with or without LY294002 were resuspended and seeded in six-well plates. The cells were cultured until confluent, and the monolayer in each was scratched using a 200 *μ*L pipette tip. The detached cells were washed using PBS. Cells were cultured with 2 mL medium at 37°C and 5% CO_2_. Photos for wounded cells were taken at 0 and 48 h under an optical microscope, and the gap distance was measured by Image J software.

### 2.10. Statistical Analysis

All data were expressed as the mean ± SD. All statistical analyses were carried out by SPSS Statistics 21.0. Student's *t*-test was applied for comparisons between two groups, while ANOVA was utilized to analyze the significant differences among multiple groups. Comparisons between NOLC1 subgroup (low vs. high) in patient survival were analyzed using the Kaplan–Meier method and assessed using the Cox regression analysis. *p* value < 0.05 was indicative of statistical significance.

## 3. Results

### 3.1. The Overexpression of NOLC1 in ESCA Tissues and Cells

**Figure 1:**
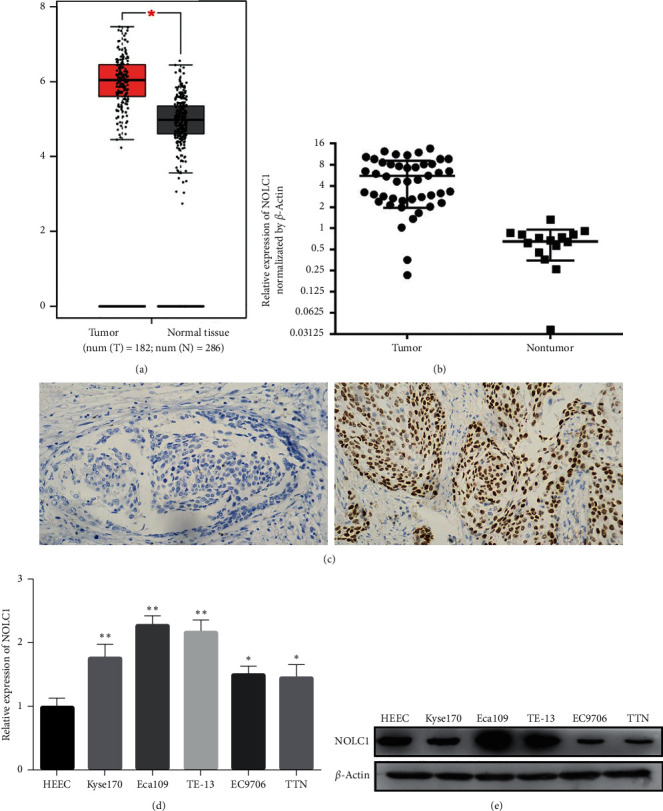
The NOLC1 expression in ESCA tissues and cell lines. (a) The expression pattern of NOLC1 in ESCA tissues and normal esophageal epithelial tissues was analyzed by GEPIA database. (b) The NOLC1 mRNA in ESCC tissues and adjacent normal tissues was measured by QRT-PCR. (c) The NOLC1 expression in ESCC tissues was evaluated by immunohistochemical staining: (1) No expression, (2) high expression. (d) The NOLC1 expression in five esophageal cancer cell lines (EC9706, Eca109, TE-13, Kyse170, T.TN) and normal esophageal cell line HEEC was detected by QRT-PCR. vs si-NC group, ^∗^*∗p* < 0.05, ^∗∗^*∗∗p* < 0.01. (e) The NOLC1 expression in five esophageal cancer cell lines (EC9706, Eca109, TE-13, Kyse170, T.TN) and normal esophageal cell line HEEC was detected by western blot.

Gene expression profiling interactive analysis (GEPIA) database was conducted to analyze the expression pattern of NOLC1 in ESCA tissues and normal esophageal epithelial tissues. The data revealed that ESCA tissues have a trend of relatively higher levels of NOLC1 expression compared with normal esophageal epithelial tissues (Figure 1(a)). In order to verify whether it was consistent with the database, the expression of NOLC1 was detected in 45 cases of ESCC tissues and 19 cases of adjacent normal tissues by QRT-PCR and immunohistochemical staining. The QRT-PCR results showed that NOLC1 mRNA was overexpressed in ESCA tissues than that in adjacent normal tissues (Figure 1(b), *p* < 0.05). The immunoreactivity of NOLC1 was observed in the brown nucleus. High NOLC1 expression was observed in ESCA tissues (55.6%) compared with normal tissues. The immunoreactivity of the low NOLC1 expression and high NOLC1 expression is shown in Figure 1(c). For further analysis, ESCA tissues were divided into 2 group on the basis of the immunohistochemical score: low NOLC1 expression group (“0” and “1+” expression of NOLC1) and high NOLC1 expression group (”2+” and “3+” expression of NOLC1). We further measured the expression of NOLC1 in five esophageal cancer cell lines (EC9706, Eca109, TE-13, Kyse170, T.TN) and normal esophageal cell line HEEC by QRT-PCR and western blot assay. QRT-PCR results showed that NOLC1 mRNA expression in five ESCA cell lines was predominantly higher than that of HEEC cells (Figure 1(d)). Consistently, western blot results indicated that NOLC1 protein levels in ESCC cell lines (Eca109 and TE-13) were notably higher than those of HEEC cells (Figure 1(e)). NOLC1 expression in Eca109 and TE-13 cells was more dramatic than that in other ESCA cell lines, so that Eca109 and TE-13 cells were utilized in subsequent experiments. These results revealed that NOLC1 was overexpressed in ESCA tissues and cell lines.

### 3.2. NOLC1 Overexpression Is Associated with Poor Prognosis of ESCA

**Figure 2:**
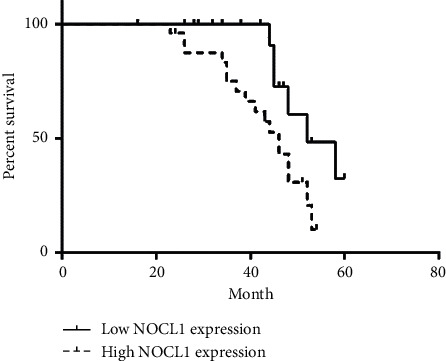
The association between NOLC1 expression and overall survival.

The relationship between the NOLC1 overexpression and the clinical parameters of the patients with ESCA is listed in Table 1. There was statistically significant correlation between the overexpression of NOLC1 and bigger tumor size (*p* = 0.027), lymph node metastasis (*p* = 0.018), and advanced TNM stage (*p* = 0.033), while there was no significant correlation between NOLC1 overexpression and patients' gender and age. Kaplan–Meier analysis revealed that patients with NOLC1 overexpression have shorter survival time compared with those with low NOLC1 expression (Figure 2, *p* < 0.01). The 5-year survival rate of ESCA patients with low NOLC1 expression was 70.0% (14/20), while that of patients with high NOLC1 expression was 32% (8/25). Univariate COX regression analysis suggested that NOLC1 was negatively correlated with overall survival (*p* = 0.029, Table 2). Otherwise, multivariate COX regression analysis demonstrated that the expression level of NOLC1 was a significant independent prognostic factor for the overall survival of ESCA patients (*p* = 0.032, Table 2).

### 3.3. NOLC1 Knockdown Inhibits the Proliferation, Migration, and Invasion and Promotes the Apoptosis of ESCA Cells

**Figure 3:**
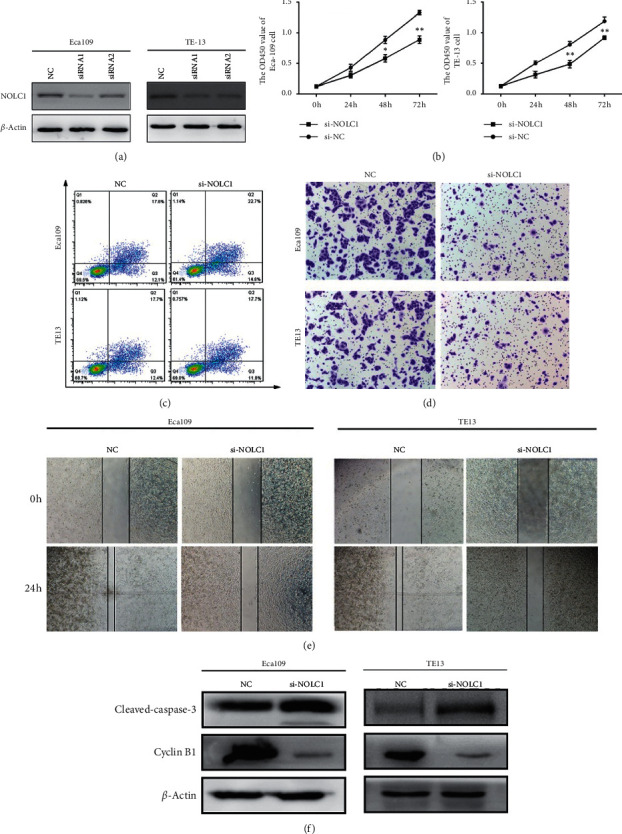
NOLC1 overexpression promotes oncogenesis and progression of ESCC. (a) The NOLC1 expression of Eca109 and TE-13 cells after NOLC1 knockdown. (b) The proliferation rate of Eca109 and TE-13 cells after NOLC1 knockdown vs. si-NC group,  ^*∗*^*p* < 0.05,  ^*∗∗*^*p* < 0.01. (c) The apoptotic rate of Eca109 and TE-13 cells after NOLC1 knockdown. (d) The invasion ability of Eca109 and TE-13 cells after NOLC1 knockdown. (e) The migration capacity of Eca109 and TE-13 cells after NOLC1 knockdown. (f) The protein level of cleaved-caspase-3 and cyclin B1 in Eca109 and TE-13 cells after NOLC1 knockdown.

In order to explore whether NOLC1 affects the development of ESCA, Eca109 and TE-13 cells were transduced with lentiviral vectors to knock down NOLC1 expression. The western blot displayed that NOLC1 expression in the sh-NOLC1-1 group and sh-NOLC1-2 group was declined compared to that in the sh-NC group (*p* < 0.05), and NOLC1 expression in the sh-NOLC1-1 group was the lowest (Figure 3(a)). Therefore, the lentiviral vectors of sh-NOLC1-1 group were selected to knock down NOLC1 in the subsequent experiments and it was named sh-NOLC1. CCK8 assays revealed that NOLC1 knockdown abrogated the proliferation of Eca109 and TE-13 cells compared to sh-NC group in a time-dependent manner (Figure 3(b), *p* < 0.05). Consistent with the impediment proliferation rate, NOLC1 knockdown encouraged the apoptotic rate of Eca109 and TE-1 cells compared to that of the sh-NC group (Figure 3(c), *p* < 0.05). Additionally, NOLC1 knockdown of Eca109 and TE-13 cells resulted in a significant impediment in invasion compared to that of sh-NC group (Figure 3(d), *p* < 0.05). Consistently, wound-healing assay revealed that the scratch in NOLC1 knockdown group of Eca109 and TE-13 cells was remarkably larger than that of the sh-NC group at 48h (Figure 3(e)). Moreover, NOLC1 knockdown depleted cyclin B1 protein level while NOLC1 knockdown elevated cleaved-caspase-3 expression. Overall, our data indicated that depletion of NOLC1 inhibits the proliferation, invasion, and migration and promotes the apoptosis of ESCA cells (Figure 3(f)).

### 3.4. NOLC1 Overexpression Promotes Oncogenesis and Progression of ESCA via Activating PI3K/AKT Pathway

**Figure 4:**
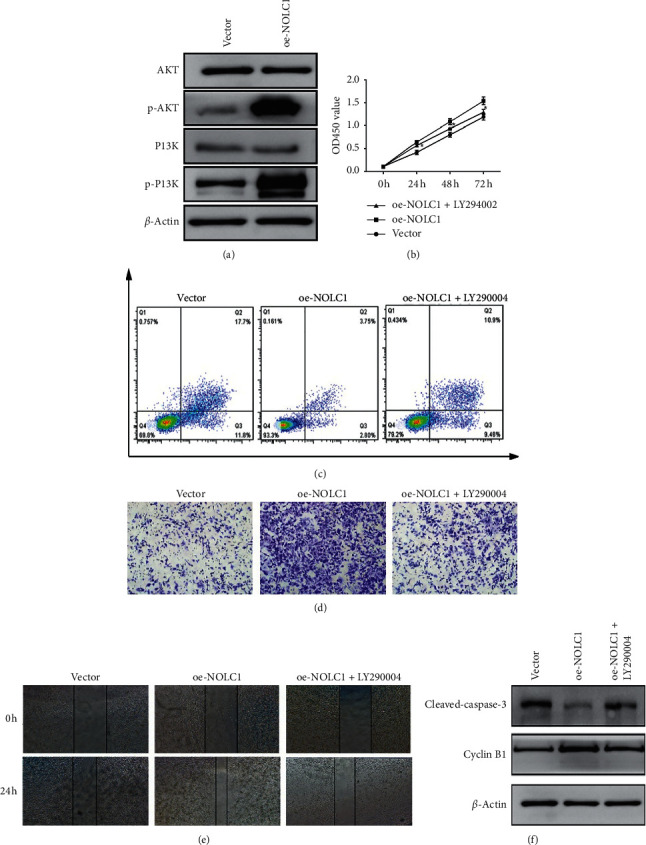
NOLC1 overexpression promotes oncogenesis and progression of ESCC via activating PI3K/AKT pathway. (a) The activation of PI3K/AKT pathway in Eca109 and TE-13 cells. (b) PI3K inhibitor LY294002 blocked cell proliferation mediated by NOLC1 overexpression vs. oe-NOLC1 group, *∗p* < 0.05. (c) LY294002 impeded apoptosis rate caused by NOLC1 overexpression. (d) LY294002 abrogated invasion ability caused by NOLC1 overexpression. (e) LY294002 abolished migration ability induced by NOLC1 overexpression. (f) LY294002 reversed the expression of cyclin B1 and cleaved-caspase-3 induced by NOLC1 overexpression.

To further investigate the mechanism by which NOLC1 exerted function on ESCA cells, we first focused on the PI3K/AKT signaling pathway, which is considered to be involved in tumorigenesis and metastasis [11, 12]. Eca109 and TE-13 cells were transduced with lentiviral vectors to overexpress NOLC1. The western blot displayed that NOLC1 overexpression elevated AKT and PI3K phosphorylation, whereas the total PI3K and AKT level remained unchanged in both Eca109 and TE-13 cells (Figure 4(a)). In order to further explore the association between NOLC1 and AKT, after NOLC1 overexpression lentivirus infection, the PI3K inhibitor LY294002 (25 *μ*mol/L) was utilized to treat Eca109 and TE-13 cells. NOLC1 overexpression significantly promoted the cell proliferation rate compared with vector group, while LY294002 deeply blocked cell proliferation mediated by NOLC1 overexpression (Figure 4(b), *p* < 0.01). Likewise, NOLC1 overexpression notably restrained the apoptosis rate of Eca109 and TE-13 cells compared with vector group, while LY294002 treatment attenuated the impediment effects on apoptosis rate caused by NOLC1 overexpression (Figure 4(c)). Additionally, NOLC1 overexpression accelerated the invasion ability of Eca109 and TE-13 cells compared with vector group, while LY294002 treatment abrogated invasion ability caused by NOLC1 overexpression (Figure 4(d)). Consistently, NOLC1 overexpression encouraged the migration ability compared with vector group in Eca109 and TE-13 cells, while LY294002 abolished the promotion effects on migration ability induced by NOLC1 overexpression (Figure 4(e)). Moreover, NOLC1 overexpression elevated cyclin B1 protein level while it depleted cleaved-caspase-3 expression. However, this phenomenon could be reversed by LY294002 (Figure 4(f)). Collectively, these bodies of evidence indicated that NOLC1 overexpression promotes oncogenesis and progression of ESCA through activating the PI3K/AKT signaling pathway.

## 4. Discussion

Deregulation of NOLC1 has been observed in various cancer types, which is associated with poor prognosis in patients with nasopharyngeal carcinoma or lung cancer. In this study, our data provided further evidence that NOLC1 expression levels were overexpressed in ESCC tissues compared to normal adjacent tumor and correlated with poor prognosis. Additionally, NOLC1 knockdown inhibited proliferation, migration, invasion, and protein expression, such as cyclin B1, and promoted the apoptosis and cleaved-caspase-3 expression of Eca109 and TE-13 cells. NOLC1 overexpression accelerated proliferation, migration, invasion, and cyclin B1 expression and inhibited the apoptosis and cleaved-caspase-3 expression of ESCA cells via activating PI3K/AKT pathway. Rescue experiments show that PI3K inhibitor (LY294002) could reverse the phenomenon caused by NOLC1 overexpression.

Targeted therapy and molecular targeted therapy are the basis of sophisticated basic medicine, leading to the improved prognosis of patients with ESCC and also leading to resurgent research interest in the research of targeted agents in ESCA. NOLC1 exerts multiple functions, such as cell differentiation regulation [13] and rRNA transcription [6]. Few pieces of research have demonstrated that it has a function correlated with tumorigenesis [14]. For example, Hwang YC et al. showed that NOLC1 is more highly expressed in nasopharyngeal carcinoma compared with normal tissues and participates in tumorigenesis together with TP53 [7]. Krastev DB et al. have further confirmed that NOLC1 can be suppressed by p53 and contributed to molecular pathways of tumor suppressor genes [15]. In addition, Huaping Huang et al. showed that NOLC1 knockdown can enhance the drug sensitivity of NSCLC chemotherapy-resistant (A549/MDR) cells to multidrug response by inhibiting cell viability and accelerating cell apoptosis [9]. However, the exact functions of NOLC1 on tumorigenesis in ESCA remain elusive. Consistent with these facts, through GEPIA database, we found high expression of NOLC1 in ESCA tissues, but the expression was comparatively weak in normal adjacent tissues. Subsequently, we detected the NOLC1 expression in ESCA tissues and cell lines. The results confirmed that NOLC1 mRNA and protein levels were abundant in both ESCA tissue specimens and cell lines. The mRNA expression of NOLC1 in cancer cells, Kyse170, ECA9706, and T.TN is different from the protein expression, which may be due to post-transcriptional regulatory mechanisms such as translation and post-translational modifications. These data by themselves are certainly not enough to explain the exact mechanism of regulation, but they do provide a direction for us to conduct more targeted experiments. Moreover, NOLC1 overexpression was associated with bigger tumor size, lymph node metastasis, and advanced TNM stage in ESCA patients. Additionally, ESCA patients with NOLC1 overexpression have shorter overall survival than that of those with low NOLC1 expression. Moreover, NOLC1 overexpression was considered as an independent poor prognostic factor affecting overall survival. Based on these pieces of evidence, it was speculated that the NOLC1 overexpression might be involved in the tumorigenesis of ESCA. To verify this hypothesis, we conducted a loss of function experiment in vitro. Results showed that NOLC1 knockdown inhibited proliferation, migration, invasion, and protein expression, such as cyclin B1, and promoted the apoptosis and cleaved-caspase-3 expression of Eca109 and TE-13 cells. Collectively, our research indicated that NOLC1 may serve as an oncogene by accelerating the proliferation, metastasis, and invasion of ESCA cells.

The specific mechanism by which NOLC1 contributed to tumorigenesis remains unclear. Intriguingly, it was recently reported that lncRNAs (TROLL-2 and TROLL-3) could counteract the interaction between WDR26 and NOLC1, while promoting the combination of WDR26 and AKT to activate the PI3K/AKT pathway in lung cancer cells, human breast carcinoma cells, and human melanoma cell lines [16]. PI3K/AKT pathway is dysregulated in various cancer cells. The PI3K/AKT pathway plays an important role in the internalization of membrane tyrosine kinases and external growth factors. Membrane kinases including epidermal growth factor receptor (EGFR) are activated by external growth factors, which initiate receptor dimerization and subsequent events to activate these intracellular pathways. AKT has multiple targets to modulate a variety of proliferative and antiproliferative signaling processes, such as survival, apoptosis, angiogenesis, cell cycle, cell-cycle energy, and DNA repair [17–22]. Additionally, Lianghai Wang et al. showed that the elevation of SOX9 inhibits the transcription of miR-203a by binding to the miR-203a promoter, thereby abolishing miR-203a-mediated impediment of PI3K/AKT/mTOR pathway [23]. Furthermore, Konstantia E. Tasioudi et al. reported that 90.5% of esophagus cancer (EC) patients expressed p-AKT mainly in the nucleus. Even in the absence of mutations in PIK3CA and Akt1, the expression of PI3K/AKT/mTOR pathway components is also expressed in EC and is associated with tumor grade, tumor stage, and clinicopathologic feature [24]. Consistent with previous research, in this study, NOLC1 overexpression elevated PI3K and AKT phosphorylation, which suggested that PI3K/AKT may be the downstream pathway of NOLC1. To verify this hypothesis, we conducted a series of rescue experiments. NOLC1 overexpression accelerated proliferation, migration, invasion, and cyclin B1 expression and inhibited the apoptosis and cleaved-caspase-3 expression in ESCA cells. Rescue experiments show that PI3K inhibitor (LY294002) could reverse the phenomenon caused by NOLC1 overexpression. Thus, NOLC1 can participate in the occurrence and development of ESCA via the PI3K/AKT pathway.

Although our research has further deepened our understanding of the function of NOLC1, this study also has some limitations that have not been clarified. However, the detailed interaction mechanism between NOLC and AKT pathway still needs to be explored, and the biological functions and mechanisms of NOLC and downstream pathways in the occurrence and development of ESCA still need further experimental exploration.

In conclusion, inhibition of NOLC1 expression may play an important role in regulating the occurrence and development of ESCA by inhibiting the proliferation, invasion, and migration, promoting the apoptosis, and regulating drug resistance-related molecules, which will provide a new perspective for the development of targeted drugs for the treatment of ESCA.

## Figures and Tables

**Table 1:** Relationship between the expression of NOLC1 and the clinicopathological variables of ESCC patients.

Variables	Case number (*n* = 45)	NOLC1 expression		*p*-value
		Low (*n* = 20)	High (*n* = 25)	
*Sex*				0.601
** **Male		8	12	
** **Female		12	13	

*Age (years)*				0.743
** **<60		9	10	
** **≥60		11	15	

*Tumor size (cm)*				0.027
** **<3		13	8	
** **≥3		7	17	

*TNM stage*				0.033
** **I + II		11	6	
** **III + Iv		9	19	

*Lymph node metastasis*				0.018
** **Yes		15	10	
** **No		5	15	

**Table 2:** Univariate and multivariate analysis of survival associated factors.

Parameters	Univariate analysis		Multivariate analysis	
	HR (95% CI)	*p*-value	HR (95% CI)	*p*-value
Sex (male vs. female)	2.011 (0.777–5.204)	0.150		
Age (<60 vs. ≥60 years)	2.333 (0.776–7.017)	0.132		
Tumor size (<3 vs. ≥3 cm)	2.239 (0.745–6.735)	0.151		
TNM stage (I + II vs. III + IV)	1.351 (0.438–4.168)	0.601		
Lymph node metastasis (yes vs. no)	2.351 (0.919–6.011)	0.074		
NOLC1 expression (low vs. high)	3.078 (1.125–8.423)	0.029	3.522 (1.114–11.141)	0.032

## Data Availability

The data used to support the findings of this study are available from the corresponding author upon request.
